# Editorial: Adipose tissue and skeletal muscle as endocrine organs: Role of cytokines in health and disease

**DOI:** 10.3389/fphys.2022.1069431

**Published:** 2022-11-23

**Authors:** Ana Claudia Garcia De Oliveira Duarte, Guilherme Fleury Speretta, Ana Maria Teixeira, Fábio Santos Lira

**Affiliations:** ^1^ Department of Physical Education and Human Motricity-DEFMH, Biological and Health Sciences Center- CCBS, Federal University of São Carlos-UFSCar, São Carlos, Brazil; ^2^ Department of Physiological Sciences, Biological Sciences Centre, Federal University of Santa Catarina, Florianópolis, Brazil; ^3^ Research Center for Sport and Physical Activity, Faculty of Sports Science and Physical Education, University of Coimbra, Coimbra, Portugal; ^4^ Exercise and Immunometabolism Research Group, Department of Physical Education, São Paulo State University (UNESP), Presidente Prudente, Brazil; ^5^ Research Center for Sport and Physical Activity (CIDAF), University of Coimbra, Coimbra, Portugal

**Keywords:** adipose tissue, skeletal muscle, adipokines, exercise trainig, health, diseases

## Introduction

One of the central factors to maintaining energy regulation is preserving the healthy functioning of white adipose tissue. An excess of this tissue is associated with metabolic dysfunctions, which promote a positive energy balance and elevated inflammatory adipokines, causing obesity and metabolic syndrome. Such disturbances in energy homeostasis can induce specific immune responses, which can influence the pathophysiology of several diseases. Thus, cytokines that promote physiological responses in the body have been examined as potential therapeutic agents for energy regulation. A significant number of these cytokines are secreted by non-adipose but metabolically active tissues, such as skeletal muscle (myokines) and the liver and may act in a corrective manner on inflammatory adipokines involved in the development of obesity. Thus, several cytokines can inhibit or suppress the actions of adipokines associated with the inflammatory obesity phenotype, as occurs with some myokines.

In this regard, the importance of physical activity cannot be understated since the benefits provided go far beyond mechanical adaptations and control of skeletal muscle energy homeostasis. The existence of humoral components such as paracrine, autocrine, and endocrine regulators that control skeletal muscle adaptive processes is also of benefit. Among the endocrine functions that are attributed to myokines, the regulation of body weight favoring a negative energy balance, the reduction of chronic low-grade inflammation, and the regulation of insulin signaling are promising for the treatment of chronic diseases such as obesity and diabetes mellitus. Recently, the number of myokines that are secreted in response to muscle contraction has been growing steadily, and new factors have been identified.

This Frontiers Research Topic includes a broad range of articles in the following areas: 1) metabolic regulations that can occur between the adipose tissue and the muscle and that are influenced by exercise; 2) the relationships between various myokines and adipokines in response to exercise; 3) metabolic disorders related to adipose tissue; and 4) mechanisms involved in the energy balance regulation process.

Therefore, knowledge of factors related to the metabolism of physical exercise, which regulates energy in the adipose tissue through interactions between metabolically active tissues, allows consolidation of this topic and may contribute to the recovery of energy homeostasis and mechanisms which, until now, have not been completely understood.

In this Research Topic, four review papers were published. Lyu et al. discussed the functions and mechanisms related to how low-level laser therapy, used for tendon repair, activates a large number of VEGF and promotes angiogenesis under hypoxia, increasing the amount of collagen type III by promoting the proliferation of fibroblasts. Throughout the remodeling phase, LLLT primarily activates M2 macrophages and downregulates inflammatory factors, thus reducing inflammatory responses. de França et al. examined the potential role of regular physical exercise as a treatment during the development of vitiligo, highlighting certain clinically relevant markers that can be analyzed in a new research avenue. de Jesus Alves et al. highlighted changes in cytokine concentrations following long-distance running and their close relationship with the running volume. The cytokines modulate compounds that play a fundamental role in the maintenance of homeostasis and cell signaling. Cai et al. discussed the ectodysplasin A/ectodysplasin A receptor system function and the physiological and pathological roles of its receptors in multiple diseases.

Two original papers discussed the effect of exhaustive exercise on immune responses. de Sousa et al. examined the course of time and the role of exercise-induced cytokines in muscle damage and repair after a marathon race. This study demonstrated that classical anti-inflammatory mediators (IL-10, IL-8, and IL-6) induced by exercise are associated with myokine response both immediately after the race and in the recovery period and may affect muscle tissue repair dynamics. Lobo et al. demonstrated that a single bout of fatiguing aerobic exercise induced similarly pronounced immunological responses in both women and men.

Three original papers demonstrated the effect of regular exercise on aging or obesity. Peres et al. explored the potential anticarcinogenic effect of plasma (*in vitro*) in older adults after exercise. The authors observed that adaptations in the blood factors of institutionalized older adults might alter cell viability and proliferation by targeting mitochondrial ROS in the prostate cancer cell line. Farinha et al. observed that both interval aerobic exercise and combined exercise programs appeared to be more effective than a continuous aerobic exercise program in decreasing chronic low-grade inflammation by mediating the production of higher levels of anti-inflammatory cytokines. However, the authors highlighted that the differences observed between the exercising groups were small and may not be clinically significant. Regarding obesity, Bonfante et al. examined the effects of the acute/chronic responses of combined training on serum pro-thermogenic/anti-inflammatory inducers and their relationship with both the nourished and fasting state in overweight type 2 diabetic individuals.

Three original papers studied experimental animal models. He et al. observed exercise-enhanced cardiac function in mice, *via* the FNDC5/Irisin-dependent mitochondrial turnover pathway, with radiation-induced heart disease. Faria et al. showed that exercise-induced melatonin potentiates increased skeletal muscle PGC-1α and optimized glycogen replenishment. Finally, da Costa et al. demonstrated that dietary intervention and moderate-intensity continuous training led to changes in the inflammatory profile of visceral adipose tissue but not in the skeletal muscle in diet-induced obese rats.

This Research Topic highlights the essential roles of adipose tissue and skeletal muscle regarding cytokines released to the bloodstream and their metabolic consequences in subjects with chronic diseases or who are healthy. Regular physical exercise leads to reduced metabolic and inflammatory disruptions and can be adopted as a nonpharmacologic treatment. Scientists are thus collaborating to find new directions for promoting a better quality of life as well as a physically active life to treat chronic disease, reinforcing that regular physical exercise can alleviate the complex conditions reported in this Research Topic.

